# Fungal DNA damage repair: from model to driver of virulence and AMR

**DOI:** 10.1042/BSR20250315

**Published:** 2026-08-07

**Authors:** Callum Parkin, Adam G. Bainbridge, Johannes Gregor Matthias Rack

**Affiliations:** Medical Research Council Centre for Medical Mycology at the University of Exeter, Department of Biosciences, Faculty of Health and Life Sciences, Geoffrey Pope Building, Stocker Road, Exeter, Devon, EX4 4QD, U.K.

**Keywords:** AMR, DSB repair, medical mycology, mismatch repair, photolyases, SSB repair

## Abstract

Maintaining genome integrity is essential for survival across all forms of life. Consequently, DNA damage response (DDR) mechanisms are evolutionarily ancient and broadly conserved. Despite their importance as major pathogens of humans, plants, and animals, fungal DDR mechanisms have primarily been studied as model systems to simplify and advance our understanding of DDRs in humans. Antifungal resistance is a major contributor to mortality from human fungal infections, which are associated with an estimated 3.8 million deaths annually. The ability of fungi to balance spontaneous production of beneficial mutations with the preservation of genomic integrity has emerged as a potential mechanism underlying the acquisition of antifungal resistance. In addition, several components of DNA damage repair pathways play direct roles in the virulence of fungal pathogens. In the present review, we provide an overview of DDR pathways, their function and conservation, and highlight specific roles in contributing to genome plasticity, adaptation, virulence, and the emergence of antifungal resistance.

## Introduction

*Fungi* represent a highly diverse kingdom that has evolved to thrive across a vast variety of environmental conditions. With an estimated 2–3 million species, fungi comprise the second largest group of eukaryotes, second only to animals in species diversity [1]. They are integral parts of many ecosystems and contribute to biodiversity in numerous ways, including nutrient cycling, improving soil structure, and aiding plant growth through establishment of mutualistic associations [2]. In humans and other animals, fungi form part of the normal microbiome, the mycobiome, and support fundamental physiological processes, including immune functions, neurological development, and nutrient acquisition [3]. Fungal pathogenicity is common in plants, with a recent genomic analysis suggesting the existence of more than 10,000 potential phytopathogenic species [4]. In contrast, fungal infections in humans are caused by a comparatively small number of species (∼300) [5,6]. Nevertheless, the global health burden is considerable: an estimated 6.5 million invasive fungal infections occur annually and are associated with approximately 3.8 million deaths, of which 2.5 million (68%; range 35%–90%) are directly attributable to these infections [7,8]. Under the ‘One Health’ framework, fungi threaten not only human and animal health but also exert major pressure on agroecosystems, thereby affecting food security, human livelihoods, and the delivery of ecosystem services [9,10].

**Figure 1:**
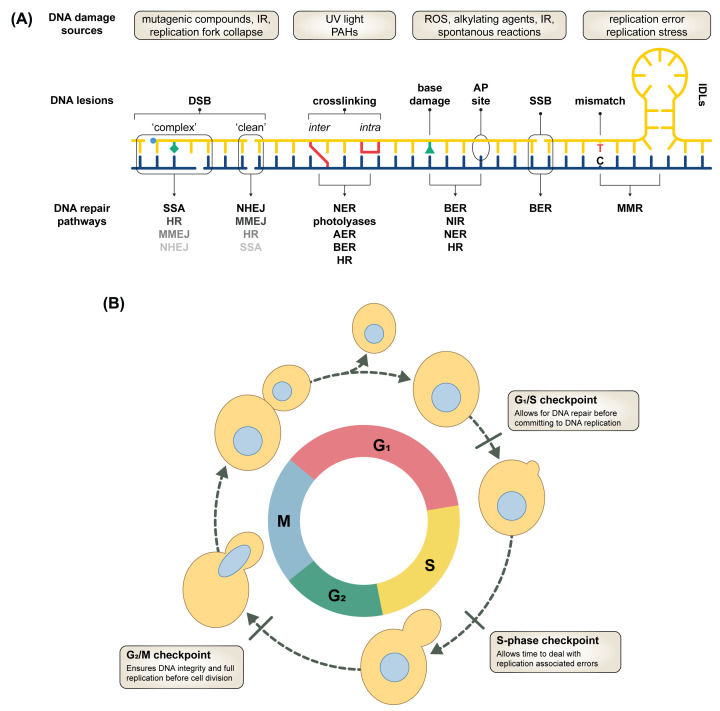
Sources of DNA damage and its molecular and cellular consequences (**A**) Schematic representation of common causes of DNA damage and their associated repair pathways. Endogenous and exogenous processes, environmental conditions, and genotoxic agents promote a range of DNA lesions that are repaired by distinct and overlapping repair pathways. Abbreviations: AER, alternative excision repair; AP, apurinic/apyrimidinic; BER, base excision repair; DBS, double-strand break; HR, homologous recombination; IDLs, insertion/deletion loops; IR, ionising radiation; MMEJ, microhomology-mediated end joining; MMR, mismatch repair; NHEJ, non-homologous end-joining; PAHs, polycyclic aromatic hydrocarbons; ROS, reactive oxygen species; SSA, single-strand annealing; SSB, single strand break, UV light, ultraviolet radiation. (**B**) Schematic of the yeast cell cycle and the major cell cycle checkpoints that can be triggered as part of the DDR.

One of the driving factors amplifying the threat posed by fungi is their ability to rapidly evolve and adapt to environmental challenges, including temperature variations, nutrient availability, transitions between environmental and host-associated niches, and antifungal treatments [11–15]. These endogenous and exogenous factors can cause numerous types of DNA lesions, which have the potential to reduce or prevent viability and require a complex network of recognition and repair proteins to reverse the damage (Figure 1A). For example, hypoxic microenvironments, as encountered by pathogens in the host, can impair mitochondrial respiration, causing electron leakage, ultimately leading to the generation of reactive oxygen species (ROS), which induces oxidative base damage [16,17]. Likewise, metabolic hyperactivation of tetraploid *Candida albicans* cells grown in glucose-rich media has been linked to elevated ROS production and increased genomic instability [18]. In addition to metabolic adaptation, DNA damage can also be induced by metabolic reaction intermediates, exposure to genotoxic agents, such as heavy metals, UV radiation, host immune defence, and antifungal treatment [18–22]. Together, these challenges can induce base alteration, like oxidation and alkylation, intra- and interstrand crosslinks (ICLs), and single- or double-strand breaks (DSBs) [23,24], which can have numerous deleterious effects on the fungal life cycle including spore inactivation, delayed spore germination, reduced mycelia extension rate, and alteration in carbon and nitrogen metabolism [25–27].

Therefore, fungal adaptability appears at first inconsistent with the observed stability of fungal genomes under standard growth conditions. This results from a low replication error rate, typically estimated at 10^−8^ to 10^−10^ substitutions per base pair per generation [28,29], combined with a relative small genome sizes, ranging from less than 3 Mbp (*Encephalitozoon cuniculi* [30]) to around 1 Gbp (*Austropuccinia psidii* [31]) and averaging 36.91 Mbp for *Ascomycota* and 46.48 Mbp for *Basidiomycota* [32]. The low mutation error rate is a result of redundant DNA damage repair systems embedded in a highly conserved signalling network, the DNA damage checkpoint system, that prevents cell cycle progression and coordinates appropriate repair (Figure 1B). The checkpoint system functions primarily during the S-phase as well as the G_1_/S and G_2_/M transitions, ensuring genomic integrity [33,34]. However, this needs to be balanced, as prolonged checkpoint activation can promote aneuploidy, copy-number variation, and loss of heterozygosity (LOH). Indeed, there are now several fascinating examples, outside the scope of the present review, that show that impaired checkpoint signalling not only influences genomic integrity but also several other fundamental cellular processes, including growth, dimorphic yeast-hyphal switching, and stress adaptation [35–39]. This suggests that checkpoint components can contribute to adaptation to host- and antifungal-associated stresses by modulating the balance between genome stability and genome plasticity. Similarly, recent work has highlighted a significant evolutionary divergence of the DNA repair gene repertoire between fungal lineages, reflecting a dynamic balance between maintaining genomic integrity and enabling adaptive flexibility [37,40–42]. While further details need to be investigated, an evolutionary divide appears to have arisen between primarily unicellular fungi, such as yeast from the *Saccharomycotina* and *Taphrinomycotina* subdivisions, and lineages with more complex multicellularity, such as *Pezizomycotina*, with yeast presenting a more limited repair gene repertoire and filamentous fungi maintaining higher repair pathways redundancies [41,42]. How this reduction can shape the evolution of fungal lineages is complex and may vary for different events, but both a generally weaker evolutionary pressure to maintain redundancy and a need to adapt to new environmental circumstances have been suggested [41,43].

Notwithstanding, DNA damage repair is not a perfect mechanism and can either fail or introduce errors, resulting in permanent genome alterations that underlie heritable adaptation. In recent years, it has become clear that the adaptation within a fungal population can be facilitated by individuals that acquire transient or stable hypermutator phenotypes [15,42,44]. These individuals are characterised by elevated mutation rates, which can increase the emergence of beneficial mutations during adaptation. Mutational changes range from single-nucleotide mutations (SNMs) and short insertions/deletions to large-scale chromosome rearrangements, including LOH and aneuploidy. This can promote the emergence of antimycotic resistance (AMR) through mutation(s) affecting a drug binding site or overexpression of the drug targets resulting from an increased genomic copy number [44,45]. However, mutations arise randomly, independently of their effect on fitness, and therefore adaptive advantages are typically accompanied by an increased burden of deleterious mutations within the population. As a result, hypermutator phenotypes may increase in frequency during periods of strong selection, when the benefits of rapid adaptation outweigh their fitness cost, but decline or be lost once adaptation has been achieved [15,44,46,47]. Similarly, hypermutators may be favoured in populations that are maladapted or exposed to unstable environmental conditions [46,47]. Such a scenario was put forward as the possible explanation for the overall high evolutionary rate associated with losses in cell cycle and genome integrity genes in yeasts of the *Hanseniaspora* genus [43]. *Hanseniaspora* can be further subdivided into a fast-evolving lineage (FEL), where these losses are even more pronounced, and a comparatively slow-evolving sister lineage. FEL strains appear to have undergone a burst of accelerated evolution and display an increased mutational load, homopolymer instabilities, and mutational signatures of endogenously damaged bases [43]. Based on the coinciding appearance of the common host species, grapes, the authors speculate that gene loss and the resulting burst in accelerated evolution helped *Hanseniaspora* to transition to this new host.

The present review provides an overview of fungal DNA damage response (DDR) mechanisms and highlights the different DNA repair pathways and sensor proteins. We particularly focus on the evolutionary diversity within the *Fungi* kingdom, the role of the DDR in environmental and host adaptation, its role in the emergence of AMR, and the potential of exploiting these pathways for novel agricultural or therapeutic interventions.

## Double strand break repair

**Figure 2:**
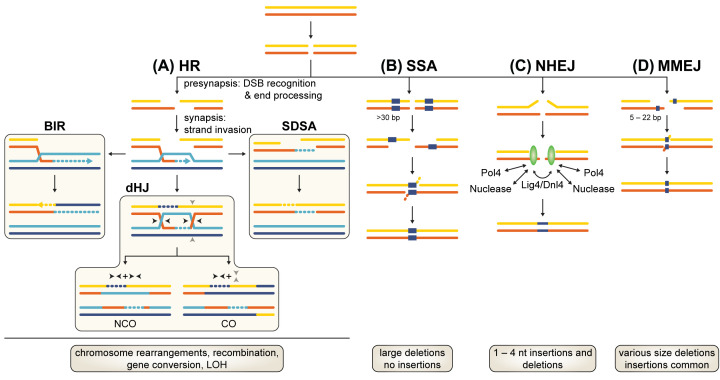
Schematic overview of the major DSB repair pathways (**A**) HR can be conceptually divided into three stages: presynapsis, synapsis, and postsynapsis. During presynapsis the DSB is recognised and the ends processed to create single-stranded DNA (ssDNA) ending in free 3′-hydroxyl moieties. Synapsis involves strand invasion into the homologous template containing at least 50–100 bp of highly similar sequence. The postsynaptic stage can be further subdivided into at least three distinct pathways (boxes): during (i) BIR, the whole distal part of the template chromatid is copied, leading to LOH. (ii) The dHJ pathway involves engagement of both strands of the DSB, either via two independent invasion events or end recapture, leading to the formation of a dHJ, which is resolved with both CO and non-crossover (NCO) outcomes possible depending on endonucleases choice (cutting sites indicated by arrow heads). (iii) In SDSA, the invading strand disengages from the D-loop after a short stretch of DNA synthesis and anneals to the second strand of the DSB, followed by gap filling and ligation. This results in local conversion without CO. (**B**) SSA requires repeat sequences in the DSB flanking regions, which are annealed, the 3′ flaps cleaved, and strands sealed by ligation. This results in loss of the sequence stretch between the repeats as well as one of the repeats. (**C**) In NHEJ, the DSB is recognised by the Ku70:Ku80 heterodimer and this facilitates recruitment of nuclease, DNA polymerase, and ligase components for end processing, if required, and break sealing. While ‘clean’ DSB can be processed error-free, ‘complex’ DSB commonly leads to short insertions or deletions. (**D**) Conceptually, MMEJ resembles SSA but is distinct from it by shorter homology requirements and protein usage. MMEJ typically results in shorter deletions, but—in contrast with SSA—may also produce insertions.

DSBs are particularly deleterious as both DNA strands are severed. Depending on the repair pathway engaged, DSB repair can lead to a variety of genomic alterations, including insertions (both templated and non-templated), deletions, co-occurring insertion–deletions and chromosome rearrangements. Mechanistically, DSB repair pathways can be categorised into those that require homology-directed DNA synthesis, referred to as homologous recombination (HR) and those relying on synapsis of broken ends and ligation, such as canonical non-homologues end joining (NHEJ), microhomology-mediated end joining (MMEJ); also termed alternative NHEJ, and single strand annealing (SSA; Figure 2). HR pathways include synthesis-dependent strand annealing (SDSA), double Holliday junction (dHJ), and break-induced replication (BIR). While yet not fully understood, pathway choice is regulated by factors, such as cell cycle phase, cell ploidy, presence of cellular stressors, and processing requirements of the DNA ends [48–51]. For example, as most fungi are haploid, HR predominates during the S/G_2_ phases, while NHEJ is active during G_1_ [51]. However, diploid and polyploid organisms, which have homologous chromosomes available during the G_1_ phase, have an increased preference for HR, resulting in increased frequencies of recombination, gene conversion, crossover (CO), and LOH events [48,52,53]. The consequences of this are not fully clear yet, but in the phytopathogenic oomycete *Phytophthora capsici*, LOH appears to drive rapid fixation of beneficial alleles and adaptability to fungicides and new hosts [54]. Moreover, evolutionary experiments with *Saccharomyces cerevisiae* suggest that both the number and types of mutations differ amongst ploidy states, with haploid cells showing a higher frequency of SNMs, whereas diploids are more prone to large-scale chromosome rearrangements [49]. One of the key decision points driving cells towards HR or NHEJ is the initial recognition of the DSB site: while binding and retention of the MRX complex favours HR, recognition by Ku70/Ku80 guides the cell towards NHEJ. By contrast, MMEJ and SSA are generally considered error-prone backup pathways that trade the ability to repair DSBs at all with the expense of accuracy and consequently, genomic stability. However, whether this is broadly applicable remains to be determined, given the diversity of DSB repair pathways across the fungal kingdom.

### Homologous recombination

HR is among the most extensively studied DSB repair systems in fungi, particularly in *S. cerevisiae*. Essential to this process are members of the radiation sensitive 52 (Rad52) epistasis group (Rad50, Rad51, Rad52, Rad54, Rdh54/Tid1, Rad55, Rad57, Rad59, Mre11, and Xrs2).

All HR pathways begin with the formation of 3′ ssDNA through 5′→3′ resection (Figure 2A). In yeast, this is mediated by the MRX complex, composed of Mre11, Rad50, and Xrs2, in collaboration with Sae2, which are rapidly recruited to DSBs [55]. The Rad50 ATPase and Mre11 nuclease form a head domain that binds to DSB, while Xrs2 provides nuclear localisation for Mre11 and assists with DNA binding [56]. The Rad50 coiled-coil domains tether DNA ends in a zinc-dependent manner [57], and while in its ATP-bound state, Rad50 dimerisation blocks the Mre11 nuclease sites and promotes DNA binding. Nuclease activity of Mre11 is subsequently activated through ATP hydrolysis, which induces a conformational change exposing the active sites, as well as interaction with phosphorylated Sae2 [58]. This creates an endonucleolytic nick that serves as an entry point for the exonuclease Exo1 and endonuclease Dna2 [56], the latter acting together with the helicase Sgs1 [59]. These factors mediate 5′→3′ resection, while MRX contributes to 3′→5′ degradation, together producing 3′ ssDNA overhangs required for HR [60]. Following resection, the ssDNA overhangs are rapidly coated by replication protein A (RPA), which protects it from degradation [61]. Subsequently, RPA needs to be replaced by the recombinase Rad51, however, the mediation of this process differs markedly amongst fungi: in *Ascomycota*, Rad52 acts as a pro-HR mediator, promoting the replacement of RPA with the recombinase Rad51, leading to the formation of the presynaptic filament [62]. In contrast, all other fungal phyla appear to carry Brh2, a homologue of human BRCA2, which can mediate Rad51 ssDNA interaction via conserved BRC element(s) [63,64]. Noteworthy, *Ustilago maydis* carries both Rad52 and Brh2 orthologues, and while stress resistance assays suggest a preference for the Brh2 utilisation partial functional redundancies between the two proteins have been observed [65,66]. Assisted by Rad54 and Rdh54/Tid1, the presynaptic complex conducts a homology search and, upon locating the homologous donor sequence, mediates strand invasion [67,68]. This forms a joined molecule, termed the displacement loop (D-loop), which serves as a recombination intermediate and provides a template for DNA synthesis. From this point in the repair process, HR can proceed through different mechanistically distinct pathways: dHJ, SDAS, and BIR (Figure 2A). In the dHJ pathway, the second 3′ overhang from the opposite side of the DSB engages with the displaced ssDNA of the D-loop. This second-end capture produces a dHJ, which is resolved by the nucleases Slx1-Slx4 and Mus81-Eme1^Mms4^ (Eme1 in fission and Mms4 in budding yeast), producing CO and NCO products [69,70]. In the SDSA pathway, the D-loop is disrupted following DNA synthesis of the first interacting strand. The extended strand is subsequently displaced from the donor template and anneals to the complementary 3′ overhang at the second DSB-end. This generates a 3′-ssDNA flap, which in yeast is removed by the structure-specific endonuclease Rad1–Rad10, yielding an NCO repair product [71,72]. Rad51-dependent D-loop formation is also a hallmark of BIR. However, in contrast with dHJ and SDSA, DNA synthesis progresses along the homologous template chromosome via a migrating D-loop (also termed the BIR replication bubble). The bubble is trailed by the newly synthesised leading strand, a long and persistent tract of ssDNA, which is filled in the last step of BIR by lagging strand synthesis. The vulnerability of ssDNA to DNA-damaging agents, together with frequent interruption of DNA synthesis due to Pol δ dissociation and an apparent reduction in polymerase proof-reading efficiency, contributes to increased levels of chromosome rearrangement and mutagenesis associated with BIR.

In contrast, SSA acts as an error-prone and tightly regulated backup mechanism (Figure 2B). In this pathway, repeat regions, exposed during DNA resection, are annealed in a Rad52-mediated process, followed by removal of non-homologous 3′ ssDNA tails and gap-filling by DNA polymerase. Since this process leads to the deletion of one repeat together with sequences between the repeats, it is considered highly mutagenic.

In addition to recombination involving allelomorphic exchanges, repetitive sequences allow for non-allele or ectopic interactions. Non-allelic HR (NAHR) can generate large-scale structural rearrangements including translocations, duplications, LOHs, and chromosomal fusions and inversions, thus contributing to genomic diversification amongst fungi whose genomes are enriched in repetitive DNA sequences, such as transposable elements (TEs) [73–76]. For example, in *C. albicans*, acquisition of fluconazole resistance was associated with the formation of an isochromosome of chromosome 5 [77]. This is thought to arise following centromeric chromosome breakage and repair via HR between repetitive DNA sequences resulting in duplication of the left arm of chromosome 5. The associated increased copy number of Erg11, which encodes the target of azole drugs, and Tac1, a transcriptional activator of multidrug efflux pumps, enhances resistance to fluconazole [78,79]. Ectopic recombination has also shaped the long-term evolution of fungal genomes. Comparative genomic analyses of the non-pathogenic species *Cryptococcus amylolentus* and the pathogenic *Cryptococcus neoformans* suggest that inter-centromeric recombination between repetitive DNA elements generated multiple chromosomal rearrangements [80]. Such events are believed to have translocated the P/R and HD mating-type loci onto the same chromosome, ultimately forming the fused MAT locus that is characteristic of pathogenic cryptococcus species.

Although HR machinery is broadly conserved across fungi, its functional consequences for virulence vary depending on the ploidy and ecological niche of the organism. For example, the basidiomycete yeasts *C. neoformans* and *U. maydis*, show increased resistance to ionising radiation (IR) compared with *S. cerevisiae* and *C. albicans* [81], and have been isolated from highly radioactive environments including the remnants of the Chernobyl Nuclear Power Plant [82,83]. In agreement with this, both *C. neoformans* and *U. maydis* utilize HR as an integral IR tolerance mechanism [81,83]. However, further studies of key regulators like Brh2 and Rad52 are required to better understand redundancies and overall physiological importance. For example, *brh2* gene loss has so far only been studies in the basidiomycete yeasts *U. maydis* and *Naganishia liquefaciens* where it phenotypically resembles *rad51* null mutants with sensitivity to IR and UV, as well as deficiencies in meiosis and HR [64,66]. However, this DNA damage sensitivity has no apparent influence on *U. maydis* virulence [84]. Another point of diversification involves the resolution of Holliday junctions: Mus81:Eme1 is an important recombination-related Holliday junction resolvase complex highly conserved across *Ascomycota* [41,85]. However, both proteins appear to be absent in *Dothideomycetes* [41,85]. This raises questions regarding the functional consequences of their absence, especially since *S. cerevisiae* *mus81* deletion mutants exhibit sensitivity to DNA-damaging agents [86], despite the presence of Yen1, which exhibits partial functional overlap with Mus81:Eme1. The sensitivity phenotype is even more pronounced in *Schizosaccharomyces pombe*, which lacks a Yen1 homologue [87,88]. These observations suggest that Mus81:Eme1 constitutes the predominant pathway for Holliday junction resolution. Consistent with this, studies using human HEK293 cells have shown that loss of Mus81 leads to formation of persistent anaphase bridges that can carry over into mitosis, ultimately promoting genomic instability [89]. Whether *Dothideomycetes* experience similar defects or whether proteins like Yen1 can compensate for the absence of Mus81:Eme1 remains unclear. Interestingly previous studies have reported frequent chromosome loss and high rates of aneuploidy within the *Dothideomycetes* [90,91] which may reflect reduced fidelity of chromosome segregation, a phenotype, in theory, consistent an absence of Mus81 and Eme1.

*Saccharomyces cerevisiae*, IR sensitivity is also a prevalent feature of Rad52 epistasis group mutants, and combining multiple mutations yields no additive phenotype [92]. However, other aspects of HR repair differ between the mutants, for example *rad59Δ* alone causes only a modest reduction in mitotic recombination, but its loss in a Rad51-deficient background reduces recombination efficiency by several orders of magnitude, highlighting the cooperative nature of HR proteins [93]. Similarly, HR-deficient *rad52Δ* mutants in *C. albicans* are hypersensitive to UV light and methyl methane sulfonate (MMS), display slow growth, and undergo spontaneous filamentation even under non-inducing conditions [94,95]. These mutants exhibit constitutive expression of hypha-associated genes and a persistent G_2_/M arrest, indicative of chronic checkpoint activation caused by the persistent presence of unrepaired DSBs. Although filamentation is a well-characterised virulence factor [96], these HR mutants have not been tested in an *in vivo* infection model; hence, their exact role of HR in virulence is unclear. In the emerging fungal pathogen *Candidozyma auris* (formerly *Candida auris*), genotoxic stress also induced the formation of pseudohyphae through activation of the S-phase checkpoint [97]. Interestingly, this phenotype is strain-dependent and might be relevant in host-imposed stressful environments during infection.

### Non-homologous end joining

Absence of a homologous template in haploid organisms during the G_1_ phase or failure to assemble the HR repair machinery necessitates repair through an alternative pathway, namely NHEJ. In yeast, NHEJ is initiated by binding of the Ku70:Ku80 heterodimer to the DSB ends, thereby protecting the termini from resection and promoting commitment to NHEJ (Figure 2C). This is followed by protein-mediated DNA end-bridging facilitated by the MRX complex, primarily through the coiled-coil domains of Rad50 and associated ‘zinc hook’ [98]. Subsequently, the NHEJ DNA ligase complex is recruited to the break. In yeast, this complex comprises of the DNA ligase Dnl4, the scaffolding protein Lif1, which are homologous to mammalian Lig4 and XRCC4, respectively, and the yeast-specific NHEJ modulator Nej1, an ancestrally related but sequentially highly diverged homologue of human XLF [99]. Recruitment of the Dnl4-Lif1 ligation machinery is mediated via protein:protein interactions between Dnl4:Ku80 and Lif1:Xrs2 complexes [100]. Moreover, *in vivo* studies have shown that Dnl4, Lif1, and Nej1 do not merely bind the Ku70:Ku80 heterodimer but also promote and stabilise the binding of Ku70:Ku80 to DNA ends [101]. This stabilisation is a crucial pathway decision point, as it inhibits the recruitment of HR-associated nucleases, thereby suppressing extensive end-resection and biasing repair towards NHEJ [101,102]. If DNA ends are not immediately suitable for ligation, minimal end processing is required to generate 5′-phosphate and 3′-hydroxyl termini and to resolve minor mismatches, overhangs, or blocked termini. In yeast, various enzymes contribute to this processing. For example, Pol4, the primary yeast NHEJ polymerase, is recruited via Dnl4 and can fill short gaps, whereas the 5′ endonuclease Rad27 removes short flap structures [103,104].

In both yeast and filamentous fungi, NHEJ repairs DSBs rapidly but often does so at the cost of fidelity by introducing insertions or deletions. Disruption of this pathway therefore eliminates mutagenic end-joining. However, the consequences of NHEJ loss for spontaneous mutagenesis under normal growth conditions cannot be generalised across fungi. For example, studies in *S. cerevisiae* showed that *ku70Δ* and *lig4Δ* mutants display fewer small-deletion mutations [105], whereas *ku70* disruption in *Yarrowia lipolytica* had no significant effect on spontaneous SNM rates, small insertions and deletions, or chromosomal rearrangements [106]. In contrast, when challenged with DNA damage stress, mutants defective in NHEJ display increased sensitivity [105,107]. Since NHEJ deficiency enhances HR efficiency without increasing spontaneous mutagenesis, disruption of *ku70*, *ku80*, or *lig4* has been widely used for HR-dependent genomic engineering, especially in haploid organisms. This strategy has been applied to model organisms such as *Aspergillus nidulans* [108], *Neurospora crassa* [109], or *Podospora anserina* [110]; human pathogens including *A. fumigatus* [111–113], *C. neoformans* [114], *Nakaseomyces glabratus* (formerly *Candida glabrata*) [115]; and in fungi of biotechnological or agricultural importance such as *Debaryomyces hansenii* [116], *Myceliophthora thermophila* [117], *Claviceps purpurea* [118], *Fusarium verticillioides* [119], *Magnaporthe grisea* [120], and *Verticillium dahlia* [121,122]. However, these strains are typically more sensitive to DNA-damaging agents, such as MMS and bleomycin, and in some cases exhibit reduced fitness relative to WT [108,109,113,123,124]. For genomic engineering, these effects can be minimised by targeting *lig4* rather than Ku proteins, which are shared with other repair pathways. Alternatively, *ku70* can be transiently disrupted during gene integration, therefore reducing the probability that subsequent phenotypes are associated with NHEJ dysfunction [125,126]. While this widespread use of Ku-deficient strains suggests that loss of Ku function is tolerated, this is not universal. In *U. maydis*, as in humans, Ku70 and Ku80 are essential for viability [127]. Beyond DSB repair, the Ku complex has been implicated in the regulation of various cellular processes such as telomere maintenance, transcription, DNA replication, and apoptosis [128]. Mechanistic investigation in *U. maydis* found that the Ku complex suppresses unscheduled DDR activation at telomeres, thereby preventing cell cycle arrest and loss of proliferation [127]. This contrasts with other basidiomycetes, like *C. neoformans* and *Coprinopsis cinerea*, where the Ku complex is non-essential [114,129] and raises broader questions about the cause of the telomere hypersensitivity in *U. maydis* and the interrelationship of DDR pathways and other cellular functions.

#### DSB repair and mobile genetic elements

In addition with classical DNA repair role, NHEJ has also emerged as an important mechanism assisting the movement of TEs. TEs are mobile genetic elements that can alter genomic architecture through insertion into new genomic locations and by promoting NAHR between dispersed TE copies [130]. This can result in structural rearrangements such as deletions, duplications, inversions, and translocations [131–133], which in turn can be major drivers of genome plasticity, contributing to adaptation and evolution through the generation of genetic diversity. The transposition mechanism of TEs involves both DNA breakage and ligation events and studies in mammalian systems have shown that repair of donor-site DSB following transposon excision as well as transposon integration follow either HR or NHEJ pathways [134,135], hence highlighting the importance of DNA repair pathways in transposition associated genome remodelling. Horizontal transfer of TEs is widespread among eukaryotes and represents an important mechanism of genome evolution. For example, horizontal transfer of TEs has been reported between rice and wheat infecting lineages of the fungal plant pathogen *M. grisea* [136]. More recently a novel class of exceptionally large TEs, termed Starships, has emerged as a driver of genome evolution in fungi. Found exclusively within the *Pezizomycotina*, Starships can be up to 700 kb pairs in length and are mobilised by a conserved tyrosine recombinase aptly named Captain [137]. Starships frequently carry diverse genes that can confer adaptive traits, including resistance to heavy metals and virulence factors such as the necrotrophic effector ToxA [138,139]. Horizontal transfer of Starships can transcend species boundaries, enabling the rapid dissemination of adaptive traits between fungal species. This may support host adaptation as closely related Starships have been documented in both plant and human fungal pathogens, where they are associated with increased virulence [137,139]. Furthermore, recent work in *Metarhizium anisopliae* has demonstrated that horizontal acquisition of a Starship element can drive extensive genome rearrangements and lead to a loss in pathogenicity, highlighting the impact Starship transposition can have on fungal genome evolution [140]. Despite their importance, the molecular mechanisms governing Starship mobility are not fully understood, and the role of DSB repair pathways in the excision and integration process remains elusive.

### Microhomology-mediated end joining

MMEJ is a DSB repair pathway that operates independently of classical NHEJ (Figure 2D). MMEJ is considered an error-prone backup mechanism, activated when the primary repair pathways, HR and NHEJ, are compromised [141]. Repair via MMEJ relies on annealing of short regions of microhomology, typically 5–22 base pairs in length, flanking the break site [142,143]. MMEJ repair can contribute to genome instability through chromosomal rearrangements [144] and, similar to SSA, can result in deletion of the sequences between the microhomologies [145].

Unlike classical NHEJ, MMEJ does not require the Ku70:Ku80 heterodimer and instead depends on DNA-end resection initiated by the nuclease activity of the MRX complex. Resection generates 3′ ssDNA overhangs that expose complementary microhomologies, enabling alignment of the broken end. The resulting non-annealed 3′ ssDNA flaps are subsequently removed by the Rad1:Rad10 endonuclease complex [142,146], gaps are filled by DNA polymerase Pol4, Rad30, Rev3, and Pol32 [146], and repair is completed by DNA ligation.

Recent genetic observations in *Cryptococcus* and *Kwoniella* indicate that MMEJ may have contributed to karyotype evolution in *Kwoniella* species, which are characterised by shorter centromeres, susceptibility to pericentric inversion, and loss of centromere function [147]. In *Cryptococcus deuterogattii*, deletion of centromere 10 showed that both neocentromere formation and MMEJ-mediated chromosome fusions can occur to restore viability [148]. These observations raise the possibility that MMEJ contributed to species survival during evolution by mediating chromosome fusion.

## Nucleotide modifications, bulky adducts, and single strand break repair

In contrast with DSBs, which occur at relatively low frequency, genomic DNA is continuously exposed to damage from endogenous and exogenous genotoxic agents as well as environmental factors such as UV light (Figure 1A). These insults can generate single strand breaks, chemical nucleotide modifications, apurinic/apyrimidinic (AP) sites as well as inter- and intra-strand crosslinks. To preserve genome integrity under these conditions, fungi rely primarily on base excision repair (BER) and nucleotide excision repair (NER) pathways, which remove oxidative, alkylation, and bulky DNA lesions. DNA damage tolerance can also be achieved via error-prone translesion synthesis (TLS), which enables the DNA replication machinery to bypass damaged templates. Interestingly, fungi possess a high redundancy in UV-induced damage repair pathways, including alternative excision repair (AER), photolyases, and components of the Fanconi anaemia (FA) pathway. Although the enzymatic mechanisms of these pathways have been well characterised in model yeasts and humans, their physiological relevance amongst the *Fungi* kingdom remains largely elusive.

### Base excision repair and nucleotide incision repair

**Figure 3:**
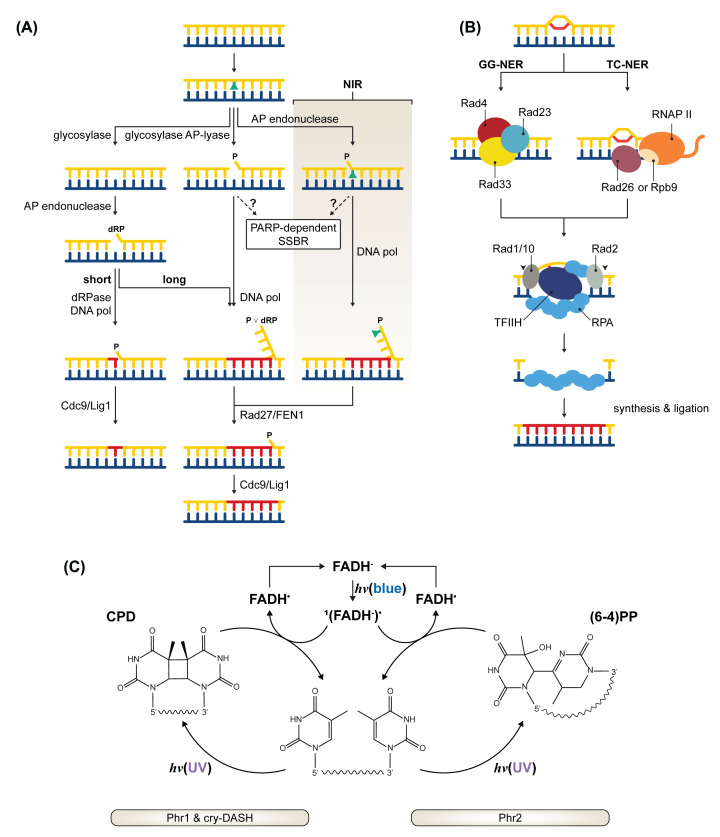
Schematic overview of pathways repairing nucleotide modifications, bulky adducts, and SSBs (**A**) Base damage is repaired in the BER pathways by damage recognition through lesion-specific glycosylases excising the damaged base to form an AP site. AP endonucleases convert the AP site into an SSB with 3′-hydroxyl and 5′-deoxyribose phosphate (dRP) ends. Alternative bifunctional glycosylase AP-lyases can directly convert damaged bases into this structure or leave only a 5′ phosphate. In short, patch repair, the 5′-dRP is converted into a 5′-phosphate, a single nucleotide is added, and the nick is sealed. In contrast, long patch BER does not require 5′-dRP conversion but the synthesis of a longer nucleotide run, followed by flap cleavage and ligation. In the nucleotide incision repair (NIR) variation, an AP endonuclease nicks the DNA 5′ of the lesion, thus allowing direct entry of the DNA polymerase for nucleotide synthesis and repair resolution in a manner similar to long patch repair. Note, the AER pathway follows NIR closely for intrastrand cross-link lesions but is initiated by the endonuclease UVDE/mus18. Abbreviations: AP, apurinic/apyrimidinic; Cdc9, cell division cycle protein 9; DNA pol, DNA polymerase; dRPase, DNA deoxyribophosphatases; FEN1, Flap structure-specific endonuclease 1; Lig1, DNA ligase 1; NIR, nucleotide incision repair; Rad27, radiation-sensitive protein 27. (**B**) NER deals with intra-strand crosslinking either via the global genome (GG-) or transcription-coupled (TC-) pathway. global genome NER (GG-NER) involves lesion recognition by Rad4–Rad23–Rad33, while in transcription-coupled NER (TC-NER) damage-stalled pol II is recognised in either a Rad26- or Rpb9-dependent manner. Both pathways lead to the recruitment of the DNA repair core of TFIIH as well as endonucleases Rad1:Rad10 and Rad2, which remove a stretch of DNA containing the damage (cut sites indicated by arrowheads). DNA across the resulting gap is subsequently resynthesis using the remaining strand as a template, and the break is sealed. Abbreviations: RNAP II, RNA polymerase II; RPA, replication protein A; Rpb9, RNA polymerase II subunit B9; TFIIH, general transcription and DNA repair factor IIH. (**C**) Simplified scheme illustrating the reaction catalysed by photolyases on the example of thymidine dimers. Briefly, the photolyase protein, which binds to DNA distorted by the presence of a pyrimidine dimer, flips the affected bases out of the double helix and into its active site. Subsequently, absorption of blue light is utilised to excite the FADH^−^ cofactor into the anionic singlet state ^1^ (FADH^−^)^•^. This is followed by two electron transfer steps: first, unto the dimer, splitting it to form two pyrimidines and a neutral flavin radical (FADH*), and second, a back transfer of an electron regenerating FADH^−^. Abbreviations: (6–4)PP, pyrimidine(6–4)pyrimidone; CPD, cyclobutene-pyrimidine dimers; cry-DASH, cryptochrome from *Drosophila*, *Arabidopsis*, *Synechocystis*, and *Homo*; FADH^−^, anionic reduced form of flavin adenine dinucleotide; Phr1/2, Photoreactivating enzyme 1/2; UV, ultraviolet.

DNA bases are susceptible to chemical modifications, including oxidation, alkylation, and deamination, either through exposure to endogenous and exogenous damaging agents or through natural decay [149,150]. For example, cytosine deamination generates uracil in DNA, while alkylation and oxidation generate modified bases such as 3-methyladenine, 7-methylguanine, and 8-oxoguanine. Although these lesions are typically small and do not distort the DNA helix, they can nevertheless impact DNA function and become mutagenic during replication, as incorrect base pairing can lead to base substitution [149,151]. Repair of such damage is carried out through the BER pathway, which involves damaged base excision, gap filling, and DNA ligation (Figure 3A).

Lesions are recognised by DNA glycosylases, a group of six structural superfamilies that acquired DNA glycosylase activities through convergent evolution: (i) uracil-DNA glycosylases, (ii) Helix–Hairpin–Helix (HhH), (iii) HEAT-like repeat DNA glycosylase, (iv) Fpg/Nei family, (v) methyl-purine DNA glycosylases, and (vi) pyrimidine dimer glycosylase superfamily. Some DNA glycosylases are bifunctional possessing both *N*-glycosylase as well as AP lyase activity, while monofunctional glycosylases catalyse only the cleavage of the *N*-glycosidic bond, generating an AP site which must be further processed by AP endonucleases [152]. The mechanism of repair-site preparation varies and may involve phosphodiesterases and DNA deoxyribophosphatases [153]. Procession through these steps generates potentially genotoxic intermediates, including AP sites, 2′-deoxyribose-5′-phosphate (dRP), and/or blocking groups at the 3′ termini. In *S. cerevisiae*, Apn1, the primary AP endonuclease, combines the glycosylase and AP lyase activities required to nick 5′ of the AP site and remove 3′-blocking groups, leaving a 3′-hydroxyl and a 5′-phosphate suitable for subsequent repair [154,155]. Following preparation of the single-strand gap, BER can proceed through one of two conserved mechanisms, termed long- and short-patch BER. In short-patch BER, which predominates in mammals, the gap is filled by a DNA polymerase and the nick sealed by a DNA ligase [156,157]. In long-patch BER, more frequent in yeasts, a longer stretch of nucleotides is synthesised by DNA Pol δ or ε starting from the 3′-hydroxyl generated by AP endonucleases [158,159]. This displaces a 5′-dRP containing flap, which is removed by the flap endonuclease Rad27/FEN1, followed by nick sealing via cell division cycle protein 9 (Cdc9)/Lig1-mediated ligation [157,160].

In a variation of the BER pathways, termed NIR, the same AP endonucleases, like Apn1, can nick DNA 5′ of the damage site, thus creating 3′-OH and 5′-phosphate termini (Figure 3A) [156,161]. Similar to long-patch BER, DNA Pol δ or ε can utilise the 3′-hydroxyl as a starting point for nucleotide synthesis. This creates a DNA-damage-containing flap that is processed via Rad27/FEN1 and Cdc9/Lig1. While very little is known in fungi, studies in other systems suggest that NIR plays primarily a role in the repair of oxidative lesions [162,163].

Although individual glycosylases differ in substrate specificity, each typically recognises a limited number of related base lesions in double stranded DNA (dsDNA) or, in both dsDNA and ssDNA [152]. The phylogenetic distribution of DNA glycosylases across the three major kingdoms is diverse. For example, fungi encode only Fpg-like enzymes of the Fpg/Nei superfamily, thus lacking homologues of mammalian NEIL1/2/3 [164]. Distribution of fungal homologues of the *E. coli* 8-oxoguanine-specific HhH-family glycosylases MutY, MutM, and MutT appears irregular. Although all *Basidiomycota* and *Ascomycota* harbour at least one homologue, repeated gene-loss events have produced distinct lineage-specific combinations: *Eurotiomycetes* encode MutM, *Candida* species (*Debaryomycetaceae* and *Metschnikowiaceae*) MutM and MutT, *Saccharomycetaceae* encode MutM, *Sordariomycetes* MutY and MutM, and *Taphrinomycotina* MutY [165]. The underlying reasons for this diversity remain unclear. However, fungi typically encode multiple glycosylases with overlapping substrate specificity. This redundancy, together with evidence for overlap with other DNA repair pathways, non-canonical glycosylase functions, cell cycle-dependent regulation, and species-specific adaptative requirements [165–168], suggests that BER participate in a complex and redundant regulatory network reflecting the evolutionary need to continually maintain efficient clearance of both endogenous and exogenous oxidative DNA lesions. Consistent with this, deletions of individual BER genes often cause subtle or no phenotypes, whereas pronounced defects generally emerge only in double or triple mutants or in combination with impairments in other repair pathways [169–171].

*S. cerevisiae* encodes a relative limited repertoire of only five DNA glycosylases: Ntg1, Ntg2, Mag1, Ogg1 (MutM), and AlkA. Ung1 removes uracil from DNA [172], Ogg1 excises oxidised bases [173], and Mag1 removes alkylated bases [174,175]. It has also been proposed that Tpa1, the AlkB-like Fe(II)/2-oxoglutarate-dependent dioxygenase, participate directly in the repair of alkylated DNA bases in yeast [176,177]. However, this role remains unclear, as Tpa1 deletion does not result in increased sensitivity to MMS [176] and recombinant Tpa1, unlike Mag1, fails to repair alkylated DNA substrates *in vitro* [176]. These observations suggest that Mag1 may possess alternative role(s) in yeast, compensating for the absence of oxidative dealkylation pathways present in other organisms.

Comparative genomics indicates that DNA repair glycosylases and endonucleases are conserved across the *Fungi* kingdom, including filamentous fungi such as *A. fumigatus*, *A. nidulans*, and *N. crassa* [164,178,179], although, these systems remain much less characterised than in model yeast. In *C. neoformans*, the DNA glycosylases Ogg1 and Ntg1 function in DNA oxidation repair. Deletion of Ntg1, but not Ogg1, increases susceptibility to DNA damage and oxidative stress, and *ntg1Δ* mutants exhibit growth defects during exposure to antifungal drugs, suggesting a role in drug tolerance [180]. In contrast with *S. cerevisiae* [181], deletion of Ogg1 or Ntg1 had no effect on spontaneous mutation rates, increasing the proportion of transversion mutations [180]. Furthermore, no phenotypic differences were observed in microevolution experiments, suggesting that BER may not contribute to short-term microevolution in *C. neoformans*.

Likewise, AP endonuclease shows important divergence across fungi. Although Apn1 shares no structural homology with the major human AP endonuclease, APE1, both enzymes catalyse analogous reactions [182,183]. In addition to the endonuclease activity, Apn1 also possesses 3′ phosphodiesterase activity, allowing removal of blocking groups at DNA ends [183,184]. In *S. cerevisiae*, knock-out of *apn1* results in sensitivity to oxidative and alkylating agents, whereas *apn2Δ* cells exhibit a near-wild-type phenotype [160]. Instead, Apn2 mainly functions in resolving topoisomerase I-derived 3′-end adducts following misincorporation of ribonucleoside monophosphates during DNA replication [185]. In addition, *apn1Δ* causes a primarily single base-pair substitution hypermutator phenotype with a 59-fold increase in A:T to C:G transversion events [186]. However, BER hierarchy differs between species: in fission yeast *S. pombe*, Apn2 rather than Apn1 performs the primary role in repairing methylated and oxidised bases, with Apn1 and the UV-specific endonuclease Uve1 acting as backups [187,188]. Similarly, Apn1 plays only a minor role in the repair of AP sites in *C. albicans* [189]. In *C. neoformans*, the roles of Apn1 and 2 appears more balanced as both individual gene deletions increase sensitivity to UV and alkylating damage, although the phenotype is more pronounced for *apn2Δ* mutants, while neither mutants shows sensitivity to oxidative stress [190]. Interestingly, inhibition of AP site resolution synergises with amphotericin B treatment in *C. neoformans*, suggesting that targeting BER may potentiate antifungal activity [190]. Environmental fungi further rely on cooperative action of photolyases and BER to ensure spore survival under UV stress, thus ensuring viability until permissive growth conditions are encountered.

### Nucleotide excision repair

NER is a highly conserved DNA damage repair pathway that removes bulky, helix-distorting lesions by excising the damaged DNA as part of an extended oligonucleotide fragment (typically 24–30 nt) [191]. NER operates through two converging sub-pathways: GG-NER, which detects and removes damage from the whole genome, including untranscribed and heterochromatic regions, and TC-NER, which targets lesions that limit RNA polymerase II (RNAP II) activity.

#### Global genome-NER

In GG-NER, lesion recognition is initiated by Rad4 (Figure 3B) [192]. The Rad4:DNA complex is stabilised and protected from proteasomal degradation by Rad23 and Rad33 [193,194]. Notably, Rad4 can recognise a wide range of chemically distinct DNA damage types [195], indicating that lesion recognition does not depend on a lesion-specific binding surface. Instead, structural analysis of Rad4 in complex with DNA suggests that Rad4 probes the tendency of the two strands to separate, which is increased upon local lesion-dependent destabilisation—a feature shared amongst NER substrates [192]. Moreover, the binding of Rad4-Rad23–Rad33 leads to local DNA unwinding and formation of an ‘open conformation’ in which the two damage bases are flipped out of the helix [192,196]. Subsequently, Rad3 and Rad25, the ATPase/helicases within the multi-subunit transcription/DNA repair factor TFIIH complex, unwind the DNA bidirectionally around the lesion, forming a DNA bubble structure [197]. In humans, the activity of both XPB (Rad25) and XPD (Rad3) is strongly inhibited by the presence of bulky lesions, leading to bubble stabilisation and recruitment of endonucleases [198]. In yeast, RPA binds the undamaged DNA strand and facilitates positioning the structure-specific endonuclease that incises the damaged DNA. The Rad1:Rad10 heterodimer introduces the 5′ incision, whereas Rad2 creates the 3′ incision, resulting in excision of a short lesion-containing DNA fragment [189,199]. During this process, PCNA is loaded onto the DNA and assists with the recruitment of DNA Pol δ to insert new complementary nucleotides, after which repair is finalised by the DNA ligase Cdc9 [200–202].

These pathways appear to be conserved amongst yeast, including *C. albicans*, where *rad1Δ* and *rad10Δ* mutants are highly sensitive to UV irradiation [189]. Interestingly, these mutants are more resistant to oxidative stress than their *S. cerevisiae* counterparts, suggesting niche-specific adaptation of DNA repair networks to different host or environmental pressures.

#### Transcription-coupled NER

In contrast with GG-NER, TC-NER is triggered by prolonged stalling of RNAP II at large helix-distorting lesions (Figure 3B). In humans, CSB, containing a SWI2/SNF2-like DNA-dependent ATPase domain, associates with CSA and UVSSA at the stalled RNAP II and facilitates recruitment of TFIIH [203]. In yeast, however, the homologue of CSA, Rad28, shows no impairment of TC-NER or UV survival. Instead, increased UV-sensitivity is observed in double mutants combining *rad28Δ* with either *rad7Δ* or *rad16Δ*, suggesting an indirect contribution to UV-tolerance [204,205]. Moreover, two partially overlapping TC-NER pathways have been described in *S. cerevisiae:* (i) a Rad26^CSB^-dependent pathways required for repair of repressed or low-transcribed genes [206] and (ii) Rpb9 (a nonessential RNAP II subunit)-dependent pathway with a more prominent role in TC-NER of highly transcribed genes [207]. This is supported by findings that both Rad26 and Rpb9 single gene deletions show only mild TC-NER impairment, while the double mutant shows severe TC-NER impairment and increased UV sensitivity [207]. While the exact mechanism is still elusive, Rad4 is required for TFIIH recruitment for both GG- and TC-NER in *S. cerevisiae* [197,208]. Despite the differences to the mammalian system in the mode of TFIIH recruitment, the two NER branches converge subsequently to complete the repair process (Figure 3B).

Noteworthy, NER appears to further diversify across yeasts [41]. For example, *S. cerevisiae* encodes a second Rad4 homologue, Rad34, associated with repair in RNA polymerase I-transcribed rDNA [209]. *S. pombe* also encodes two Rad4 homologues (termed Rhp4A/B) which are linked with repairing cyclobutene-pyrimidine dimers (CPDs) in transcribed (Rhp4A) and non-transcribed regions (Rhp4B) [210]. In contrast, in entomopathogenic fungi, such as *Beauveria* and *Metarhizium*, NER appears to play more of a secondary role and is closely integrated with the activity of light-dependent photolyases (see below) [211]. However, most of these studies focus on conidial UV resistance, a key factor for the application of these fungi as insecticides, and the role of NER in later life stages needs further investigation.

### Photolyases

In addition to NER, many fungi utilise photoreactivation as an alternative mechanism to repair UV-induced DNA lesions, with CPDs and pyrimidine(6–4)pyrimidone (6–4PPs) representing the most common photoproducts [212]. Both CPDs and 6-4PPs are covalent pyrimidine adducts that distort the DNA helix, therefore interfering with replication, transcription, and other essential DNA-dependent processes [213]. Cryptochrome/photolyase family (CPF) proteins are flavoproteins with a variety of physiological functions: (i) blue light-sensing photoreceptors that regulate development and circadian processes in plants and animals, (ii) light-independent transcriptional regulators, and (iii) blue light-activated photolyases that reverse UV-induced DNA damage via photoreactivation. The latter is an evolutionarily ancient repair mechanism that requires only a single CPF protein that both recognises and reverses the DNA damage, thus contrasting the elaborate protein assemblies of other repair pathways [214]. Photolyases are activated by blue light and act on distinct lesions in a type-specific manner: Phr1 and cry-DASH (after genera in which this cryptochrome/photolyase subfamily were first identified: cryptochrome from *Drosophila*, *Arabidopsis*, *Synechocystis*, and *Homo*) target CPDs, whereas Phr2 targets 6-4PPs (Figure 3C). To mediate UV-damage repair, photolyases scan the genome for pyrimidine dimers, and upon lesion recognition, the damaged bases are flipped out of the DNA helix into the enzyme’s active site [215], while maintaining the integrity of the DNA backbone. Absorption of blue light excites the bound reduced cofactor FADH^−^ into a high-energy state, allowing forward electron transfer to the cross-linked base pair and generation of a radical semiquinone intermediate ^1^(FADH^−^)^•^. In CPDs, the transferred electron promotes the stepwise splitting of the crosslinking bond, while repair of 6-4PPs requires an additional proton transfer from a photolyase histidine residue [216]. In both cases, the reaction cycle ends by back-transfer of an electron to regenerate FADH^−^. As this mechanism does not involve excision or resynthesis of DNA, photoreactivation is inherently error-free, ensuring high-fidelity repair of UV-induced lesions.

Photolyases have been identified across a large spectrum of organisms including bacteria, fungi, plants, invertebrates, and a subset of vertebrates, but appear to have been lost during human evolution [217,218]. Functional studies in fungi such as *N. crassa* and *Botrytis cinerea* showcased the importance of photolyases in UV-induced DNA damage resistance [219,220]. In *Trichoderma atroviride*, knockout of Phr1 abolishes the photoreactivation of UVC-treated spores [221]. In entomopathogenic fungi, Phr-type photolyases appear to be the dominant form of UV-induced DNA damage repair [211]. For example, in *Beauveria bassiana*, individual deletions of Phr1 or Phr2 reduce conidial tolerance to UVB radiation [222], accompanied by increased accumulation of CPDs and 6-4PPs. Interestingly, partial compensation between Phr1 and Phr2 was observed, indicating a degree of redundancy between the two major photolyase enzymes in this system. In addition, the *B. bassiana* Rad23 ortholog does not participate in NER, as described in other organisms, but rather interacts with Phr2, enabling repair of CPD lesions [223]. In contrast, in pathogenic yeasts, including *C. albicans* and *C. neoformans*, the functional roles of photolyase-like genes remain poorly defined, and UV-repair capacity appears to rely predominantly on NER and checkpoint signalling rather than classical photoreactivation [224]. Similarly, the function of cry-DASH appears to have diversified within the fungal kingdom: in *Mucorales* species, Phr1/2-type photolyases are absent and cry-DASH-like photolyases play a crucial role in CPD reversal [225,226]. In contrast, cry-DASH cryptochromes in other fungi, including *N. crassa*, *U. maydis*, and *Fusarium fujikuroi*, have lost their DNA repair ability and act as photoreceptors [227–229].

### Alternative excision repair

An additional mechanism for the repair of UV-induced DNA lesions, acting independently of both NER and photolyases, is AER. In this pathway, the DNA damage is recognised by UV damage endonuclease (UVDE; Uve1 in *S. pombe*), which cleaves 5′ to the damage site generating a free 3′-hydroxyl group as a substrate for repair synthesis by DNA Pol δ through strand displacement [230,231]. The flap endonuclease Rad2 subsequently removes the displaced strand, allowing ligation to take place [230,232]. Genome analysis and UV-stress experiments suggest that UVDEs are conserved in filamentous fungi, including *A. niger* and *N. crassa*, but are absent in entomopathogenic fungi [178,233,234]. Although AER can theoretically remove 6-4PPs and CPD more rapidly than NER [235,236], simultaneous disruption of both AER and NER sensitises cells synergistically to UV damage. This highlights that AER is NER-independent and may act as a backup mechanism, consistent with findings that NER is the more efficient and commonly utilised repair system. One possible explanation is that AER introduces additional DNA nicks, which may interfere with the replication process [237,238]. Furthermore, biochemical studies in *S. pombe* indicate that AER has a broader substrate specificity than first thought, including the ability to remove non-UV-induced DNA adducts, such as AP sites, and to participate in mismatch repair (MMR) [239,240]. In *C. neoformans*, UVE1 is regulated by the white collar complex, a two-protein complex comprised of a photoreceptor and a transcription factor that regulates several key processes, such as circadian rhythms and metabolism in a light-dependent manner. *Cn*UVE1 localises to the mitochondria, where it contributes to mitochondrial genome repair and defence against UV damage [241]. Intriguingly, *Pseudogymnoascus destructans*, the causative agent of the white-nose syndrome in bats, has lost UVE1, while non-pathogenic *Pseudogymnoascus* species have maintained the gene [242]. This loss causes a severe UV sensitivity [242], but might contribute to the observed ability of *P. destructans* to maintain phenotypic diversity and rapidly adapt, thus supporting persistence following its recently introduction to North America from a native Eurasian population [243,244].

### Emerging pathways in fungal DNA repair

The DNA strand and base damage repair pathways described above are generally well characterised, and current research increasingly focuses on lineage-specific differences in their utilisation and regulation. In contrast, other DDR pathways first characterised in mammals, including the FA pathway and DNA damage-induced ADP-ribosylation signalling, remain understudied in fungi. Nevertheless, recent work suggests that these pathways may play important roles in DNA repair and virulence, at least in certain fungal lineages.

#### Fanconi anaemia pathway

ICLs are DNA lesions where two strands of the double helix become covalently linked, thereby blocking replication and transcription. They can arise endogenously from reactive metabolites, including nitrous acids and aldehydes, or be induced by chemotherapeutic agents such as cisplatin [245].

In higher eukaryotes replication-coupled ICL repair is mediated via the FA pathway. The FANCM–MHF1–MHF2 complex, in conjunction with UHRF1, recognises and binds to ILC sites [246,247] and subsequently recruits the FA core complex. The monoubiquitin ligase activity of the core complex is activated through phosphorylation by the checkpoint kinase FANCM, leading to monoubiquitination of FANCD2 and FANCI and formation of the ID complex [248]. This complex coordinates the recruitment of downstream effectors, including endonucleases, scaffold proteins, and other repair factors, enabling cross-link unhooking, translesion DNA synthesis, and HR-mediated repair [249,250]. The pathway is ultimately terminated by deubiquitination of FANCD2 and FANCI by the USP1:UAF1 complex [249]. In *S. cerevisiae*, homologues of several FA-related proteins have been identified, including CENPS and CENPX (MHF1/2 homologues) and MPH1, CHL1, and SLX4 (homologues of FANCM, FANCJ, and FANCP) [251,252]. However, both ID complex components are absent in *Ascomycota* and *Basidiomycota*, suggesting that the FA pathway is incomplete in *Dikarya* [248]. Consistently, yeast deficient in these homologues do not show major defects in ICL repair [252] and instead primarily repair ICLs via a Pso2-dependent pathway, in which the exonuclease activity of Pos2 is stimulated by the RecQ4-related helicase Hrq1, allowing lesion processing and access for translesion polymerases [253]. Notwithstanding, some studies suggest that FA homologues might still play an overlapping or redundant role alongside the Pso2 pathway in *S. cerevisiae* [251,252,254].

In contrast, early-diverging fungal lineages appear to retain a more complete FA-related repertoire, with homologues of up to 32 FA-associated proteins identified in fungal proteomes [248]. For example, *Mucor lusitanicus* is predicted to encode multiple FA core (FANCA, FANCE, FANCL, and FANCM), and activation proteins (MFH1 and MFH2), one ID component (FANCD2) alongside the monoubiquitination factor UBE2T, and several downstream repair factors. Most predicted fungal FA proteins cluster closely with their animal counterparts, although SLX4 and FANCJ homologues show divergence in domain architecture. Notably, FANCJ homologues in early-diverging fungi lack the canonical DEAD-box helicase domain, and those in *Olpidiomycota* and *Microsporidia* are highly truncated, lacking the C-terminal RAD3-like helicase domain, which may affect function [248].

Together, these findings suggest that a minimal FA pathway may persist in ancient fungal lineages such as *Mucoromycota*, with progressive gene loss and pathway reduction occurring during evolution toward *Dikarya* [248]. However, functional studies assessing the roles of these retained FA proteins in lineages such as *Mucoromycota*, *Glomeromycota*, and *Chytridiomycota* require further exploration.

#### ADP-ribosylation signalling

ADP-ribosylation is conserved in all kingdoms of life, as well as in some viruses, and regulates many fundamental cellular processes, including transcription, metabolism, cell cycle regulation, and inter-microbial warfare. It is also known as a master regulator of multiple mammalian DNA repair pathways, including SSB repair, HR, and MMEJ/NHEJ [255–259]. The modification is established by PARPs, which cleave NAD^+^ and transfer the ADP-ribose moiety onto various targets, including proteins and nucleic acids [258].

In Fungi, PARPs have been lost from species with a primary yeast-like morphotype, including important pathogens such as *Candida*, *Pneumocystis*, and *Cryptococcus* species. However, they are typically retained in primarily filamentous dimorphic and filamentous fungi, including major animal and plant pathogens such as *Aspergillus*, *Batrachochytrium*, *Histoplasma*, *Fusarium*, and *Magnaporthe* [260,261]. In contrast with mammals, which possess three DNA repair-associated PARPs, fungi contain only a single homologue, and the absence of redundancy might contribute to the importance of fungal PARPs for virulence. Consistent with this, studies in *A. fumigatus*, *M. oryzae*, and *Fusarium oxysporum* showed that deletion of *parp* reduced fungal virulence and improved host survival [260,262,263].

We recently showed that activation of *A. fumigatus* PARP1 relies on the recognition of 5′-phosphorylated DNA ends in the context of nicked, gapped or 3′ overhanging DNA, but not blunt end substrates [260]. Surprisingly, and in contrast with other systems, the fungal BRCT domain forms part of the multidomain assembly, which contributes to DNA damage binding and increases catalytic efficiency. Despite these mechanistic insights, the molecular pathway(s) linking ADP-ribosylation with fungal pathogenicity remains elusive. Virulence attenuation in *Magnaporthe* was suggested to occur through ADP-ribosylation of 14-3-3 proteins, therefore influencing appressorium formation [262], while in *Fusarium*, ADP-ribosylation of protein disulfide isomerases is thought to influence ER homeostasis and pathogenicity [263].

The identified virulence roles of fungal PARPs across multiple species and divergence from the mammalian pathway of PARP regulation suggest ADP-ribosylation could emerge as a promising strategy for the development of novel antifungal therapies.

## Mismatch repair

**Figure 4:**
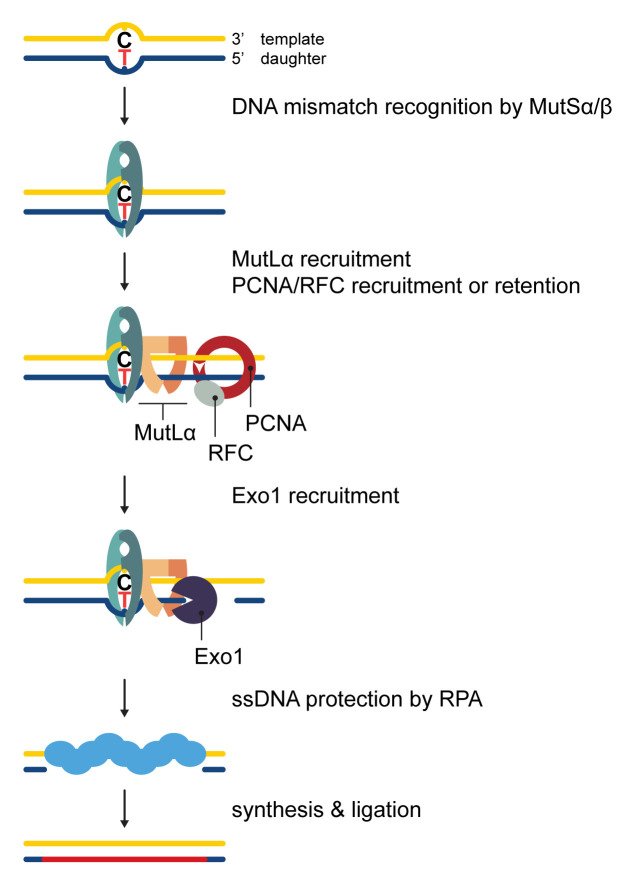
Schematic overview of the MMR pathway Recognition of a base mismatch by MutSα (Msh2–Msh6) or MutSβ (Msh2–Msh3) and MutLα (Mlh1–Pms2) results in the formation of a ternary complex. This recruits other MMR factor, including PCNA and RCF, and stimulates Pms2’s endonuclease activity, resulting in a DNA nick (white arrowhead). This provides an entry point for Exo1, which removes the mismatch base from the daughter strand. DNA across the resulting gap is subsequently resynthesised using the remaining strand as a template, and the break is sealed.

During normal DNA replication, lesions can arise through inaccurate activity of DNA polymerases (Figure 1A). The DNA MMR pathway is one of the most crucial genome-maintenance systems in eukaryotes, correcting single-base mismatches and small insertion–deletion loops that escape polymerase proofreading. Polymerase proofreading provides a first checkpoint of replication fidelity, and its combination with the MMR pathway increases overall accuracy by several orders of magnitude (Figure 4) [264]. In yeast, mismatches are first detected by heterodimers of the MSH protein family. The Msh2:Msh6 complex (MutSα) primarily recognises base mismatches and small insertion–deletion loops of 1–2 nucleotides, whereas the Msh2:Msh3 complex (MutSβ) has preference for larger loops, up to approximately 17 nucleotides [265]. Upon mismatch recognition, the MSH complex exchanges ADP for ATP, forming a clamp-like structure that can dissociate from the lesion and slide along the DNA strand [266]. The MSH complex then recruits additional repair proteins including PCNA, RFC, and the Mih1:Pms1 heterodimer (MutLα) [267,268]. PCNA stimulates the activity of Mih1-Pms1, which introduces a nick into the newly synthesised DNA strand, creating an entry point for the exonuclease Exo1, which excises DNA in 5′→3′ [265,269]. Finally, DNA polymerase can resynthesise the excised region, restoring the correct sequence. Consistent with their central role in replication fidelity, loss of Msh2 or Pms1 in *S. cerevisiae* results in elevated base substitutions, microsatellite instability, and increased recombination between divergent sequences [270].

Defects in MMR create hypermutator phenotypes, in which mutation rates are dramatically elevated relative to wild type [45]. Such strains have been identified in multiple pathogenic fungi and often exhibit enhanced adaptive capacity under antifungal pressure. *Nakaseomyces glabratus* provides one of the clearest examples, as more than half of clinical isolates carry nonsynonymous mutations in *Msh2* [271], many of which compromise MMR function. Despite lacking morphological virulence traits typical of *Candida* pathogens, such as hyphae formation [272], *N. glabratus* compensates through an exceptional capacity for microevolution. Accordingly, MMR defects are likely major contributors to its clinical persistence and frequent acquisition of antifungal resistance.

In *C. neoformans*, hypermutator isolates are less common but have been identified among clinical isolates [264,273], and some exhibit mutation rates up to ∼200-fold higher than the laboratory strain KN99α [264]. Genetic analyses in *C. neoformans* indicate that deletion of any single core MMR gene, either *Msh2, Mlh1*, or *Pms1*, is sufficient to fully disrupt the pathway, as double mutants do not show additive effects in mutation rate [264,274]. Interestingly, cryptococcal *msh2Δ* strains show minimal sensitivity to DNA-damaging agents, implying that MMR is not critical for acute stress resistance but is instead essential for shaping long-term genomic diversification and adaptation.

Across fungal pathogens, MMR deficiency generally has modest immediate effects on growth and survival under genotoxic stress, as seen in *C. albicans*, *N. glabratus*, and *C. neoformans* [264,275]. However, experimental evolution studies reveal substantial consequences over time. *C. neoformans msh2Δ* and *mlh1Δ* strains accumulate mutations rapidly and develop diverse phenotypic changes after only a few passages, with *msh2Δ* backgrounds showing particularly high variability [264]. In contrast, *pms1Δ* strains exhibit little phenotypic variation, possibly because deleterious mutations accumulate to a level incompatible with survival, effectively purging variation from the population. Notably, only *pms1Δ* strains display attenuated virulence in murine infection models [264], suggesting MMR loss is tolerated within a narrow mutational window until fitness substantially decreases.

In *A. fumigatus*, the consequences of MMR deficiency differ again. Deletion of the Msh2 homologue (*mshAΔ*) does not confer sensitivity to DNA-damaging agents and can increase resistance to the UV-mimetic compound 4-NQO [276]. Both *mshAΔ* strains and clinical isolates with deleterious *mshA* mutations show reduced virulence in murine infection models, but intriguingly, virulence is restored after several rounds of asexual division when tested in *Galleria mellonella*, suggesting compensatory mutations can offset the initial fitness cost. Recent work examining *msh6*, *mlh1*, and *pms1* reveals that MMR deficiency accelerates acquisition of azole resistance, and that some MMR mutations, such as *msh6* G223A, are globally distributed, carry little or no fitness cost, and are strongly associated with the major azole-resistance allele *cyp51A* TR34/L98H [277,278]. These findings suggest that environmental hypermutator strains may serve as reservoirs for clinical resistance, enabling rapid evolution of resistance to both established and newer antifungal classes.

Although MMR is broadly conserved across *Ascomycota*, several plant-pathogenic fungi, including *Erysiphe* and *Blumeria* species, have independently lost multiple MMR genes [279]. These species exhibit proliferation of mononucleotide runs, elongation of microsatellites, and accelerated mutation rates consistent with long-term hypermutation. However, functional analyses in plant pathogens remain limited, leaving a significant knowledge gap in understanding how MMR shapes pathogen evolution in agricultural settings.

Overall, MMR dysfunction dramatically increases mutation rates and accelerates adaptive evolution in fungal pathogens. Although hypermutation is often associated with moderate fitness costs, it may become advantageous in fluctuating or stressful environments, such as during antifungal treatment, where beneficial mutations are strongly selected. This mirrors observations in bacteria, where MMR-defective strains persist in chronic infections such as cystic fibrosis lung disease [280] and where mutator alleles increase in frequency through hitchhiking with beneficial mutations [281]. Taken together, these studies illustrate how loss of MMR shifts the evolutionary trajectory of fungal pathogens, promoting rapid emergence of antifungal resistance and increasing phenotypic diversity during infection.

## Translesion DNA synthesis

Stalling of high-fidelity DNA polymerases during replication causes replication stress, which can lead to helicase-polymerase uncoupling and potential fork collapse. To prevent this, cells utilise TLS as a DNA damage tolerance mechanism. TLS allows synthesis through the DNA lesion, such as damaged bases, UV adducts, and AP sites, by switching from the proofreading polymerases to specialised TLS-polymerases [282–286]. TLS has been extensively studied in *S. cerevisiae*, where the E2 ubiquitin enzyme Rad6 and its cognate E3 ubiquitin ligase Rad18 are fundamental components. Rad6 is associated with the repair of bulky lesions, as loss of Rad6 catalytic activity results in hypersensitivity to UV radiation as well as alkylating and DNA cross-linking agents [286]. TLS is initiated upon replication fork stalling through monoubiquitination of PCNA at Lys164 by the Rad6–Rad18 heterodimer [287]. It is not entirely resolved whether PCNA ubiquitination is a TLS initiation signal, strengthens interactions with TLS polymerases, and/or facilitates polymerase switching. Following PCNA monoubiquitination, the replicative polymerase (Pol α, δ, ε) is replaced by a TLS-polymerase (Pol ζ, η, ι, κ). These polymerases lack intrinsic exonuclease activity, thus allowing base insertion opposite damaged bases without proofreading activity. In addition, they typically possess enlarged active sites capable of accommodating chemically modified and mismatched bases [288–290]. The TLS-polymerases synthesise approximately 18 nucleotides past the lesion, bypassing detection by proofreading polymerases. Subsequently, PCNA is de-ubiquitinated, and the TLS-polymerase dissociates to be replaced by a high-fidelity counterpart [288].

In fungi, the biological consequences of deletion of Rad6-associated pathways are context-dependent. In *N. glabratus*, deletion of *Rad6* has been linked to increased biofilm formation and reduced ROS production during macrophage co-incubation, promoting fungal survival and enhancing virulence in a murine model [291]. Similarly, deletion of Rad18 in *C. albicans* increases virulence in *Galleria mellonella* infection models and enhances intracellular survival within macrophages. Transcriptomic analyses indicate that Rad18 up-regulates key virulence genes, including Hwp1 and Ece1, which are involved in binding to host epithelial cells and candidalysin production, respectively [292–294].

In contrast, Rad6-mediated monoubiquitination pathways are essential for infection-related development and pathogenicity in *M. oryzae* [295]. In *B. bassiana*, Rad6 deletion results in growth defects in response to multiple DNA-damaging agents and attenuates virulence [296]. Transcriptomic analysis of the *B. bassiana* Rad6 deletion revealed up-regulation of DNA repair genes under non-stress conditions but a shift toward metabolic pathways upon oxidative stress exposure, with DNA repair pathways no longer represented among the top enriched KEGG categories [296]. Collectively these findings indicate Rad6-associated ubiquitination pathways play crucial, but context-dependent, roles in fungal virulence and antifungal drug responses. However, the molecular mechanisms by which Rad6-mediated ubiquitination and DNA repair cause these phenotypes remain poorly defined.

On the other hand, a recent study indicated that arbuscular mycorrhizal fungi of the *Glomeromycotina* as well as *Microsporidia*, obligate intracellular parasites of animals, have undergone reductive evolution of their replicative and translesion polymerase repertoire [297]. These losses are associated with an increased mutation rate, hence genetic variability, and the authors suggest this may aid adaptation to the host and shape the evolution of these lineages.

## Conclusion and outlook

The fungal DNA repair field has long focused on yeasts as model organisms to understand mammalian DNA repair mechanisms. However, accumulating evidence indicates that fungal DNA repair is a rich and diverse area of research in its own right, with a high degree of divergence not only from the mammalian pathways but also across the fungal kingdom. Although we have obtained preliminary insights, especially for the MMR pathway, our understanding of how fungi utilise DNA repair to drive adaptation, pathogenicity, and AMR remains limited. This is especially true for filamentous and early-divergent fungal lineages, where knowledge of DNA repair systems is still in its infancy. Moreover, several repair pathways, including AER, FA, and ADP-ribosylation-mediated mechanisms, are markedly understudied. Further exploration of the fungal DDR will be important for better understanding how DDR pathways integrate and influence other cellular processes, particularly those governing in-host survival and pathogenicity as well as evolutionary processes driving DNA repair pathway diversification and adaptation. In this context, comparative transcriptomic analyses and other systems-level approaches are likely to be instrumental in advancing our understanding of the underlying regulatory mechanisms. Notably, the transcriptional response to DNA damage can substantially differ from the gene expression patterns previously reported in yeast and other model organisms [41,151,235,298–300]. These observations underscore the importance of broader comparative analyses to determine the extent to which DNA damage responses are conserved or species-specific and to identify the molecular basis of these differences. Insights into these are particularly relevant for our understanding how fungi adapt to shifting environmental pressures, including climate change and the use of clinical and agricultural antifungals. A deeper understanding of the fungal DDR may therefore not only advance fundamental biological knowledge but also offer opportunities for novel therapeutic or agricultural interventions aimed at safeguarding plant, animal, and human health.

## Abbreviations

AER: alternative excision repair
AMR: antimycotic resistance
AP: apurinic/apyrimidinic
BER: base excision repair
BIR: break-induced replication
Cdc9: cell division cycle protein 9
CO: crossover
CPF: cryptochrome/photolyase family
CPDs: cyclobutene-pyrimidine dimers
cry-DASH: cryptochrome from *Drosophila*, *Arabidopsis*, *Synechocystis*, and *Homo*
DDR: DNA damage response
dHJ: double Holliday junction
D-loop: displacement loop
dRP: 2′-deoxyribose-5′-phosphate
DSBs: double strand breaks
dsDNA: double stranded DNA
FA: Fanconi anaemia
FEL: fast-evolving lineage
GG-NER: global genome NER
HhH: Helix–Hairpin–Helix
HR: homologous recombination
ICLs: interstrand crosslinks
IR: ionising radiation
LOH: loss of heterozygosity
MMS: methyl methane sulfonate
MMEJ: microhomology-mediated end joining
MMR: mismatch repair
NAHR: non-allelic HR
NCO: non-crossover
NER: nucleotide excision repair
NHEJ: non-homologues end joining
NIR: nucleotide incision repair
RNAP II: RNA polymerase II
ROS: reactive oxygen species
RPA: replication protein A
SDSA: synthesis-dependent strand annealing
SNMs: single-nucleotide mutations
SSA: single strand annealing
ssDNA: single stranded DNA
TC-NER: transcription-coupled NER
TEs: transposable elements
TLS: translesion synthesis
UVDE: UV damage endonuclease
